# Complete mitochondrial genome of the bamboo-shoot fruit fly, *Acrotaeniostola dissimilis* (Diptera: Tephritidae) and its phylogenetic relationship within family Tephritidae

**DOI:** 10.1080/23802359.2019.1694455

**Published:** 2019-12-11

**Authors:** Ping-Fan Jia, Jian-Hong Liu, Wen-Li Dan

**Affiliations:** a College of Life Science, Shangrao Normal University, Shangrao, Jiangxi, China;; b Key Laboratory of Forest Disaster Warning and Control of Yunnan Province, Southwest Forestry University, Kunming, Yunnan, China

**Keywords:** *Acrotaeniostola dissimilis*, mitochondrial genome, molecular phylogeny

## Abstract

*Acrotaeniostola dissimilis* (Diptera: Tephritidae) is an insect pest of bamboo shoot and distributed in the Yunnan and Sichuan Provinces, Southwest China. Complete mitogenome sequence has been determined in this study. The circular genome is 15384 bp long and contains a standard gene complement, that is, the large and small ribosomal RNA subunits, 22 transfer RNA genes, 13 genes encoding mitochondrial proteins, and a non-coding A + T-rich control region. The phylogeny showed that *A. dissimilis* in tribe Gastrozonini was a monophyletic branch and clearly separated from both tribe Dacini and tribe Ceratitidini with high bootstrap value supported.

Members of genus *Acrotaeniostola* selected bamboo shoots as breeders (Hardy 1988). *Acrotaeniostola dissimilis* is also an insect pest of bamboo shoot and distributed in the Yunnan and Sichuan Provinces, Southwest China (Hancock and Drew 1999). At present, there are no mitochondrial genome data in genus *Acrotaeniostola* of family Tephritidae in Genbank. In this study, we first reported the complete mitogenome of *A. dissimilis*. The results would be helpful for phylogenetics, population genetics, and exact species identification.

Female flies were caught from Kunming city (25.05°N, 102.76°E), Southwest China, on 11 May 2018. Specimens were deposited in the museum of Southwest Forestry University (Voucher KM20180511), Kunming, China. Genomic DNA was extracted according to the manufacturer’s instruction in the DNeasy Blood and Tissue kit (Qiagen, Hilden, Germany) and then sequenced using Illumina’s HiSeq2000 platform (Illumina, San Diego, CA, USA). The sequence was preliminarily aligned within the CLUSTAL X program in BioEdit software. Both protein-coding genes (PCGs) and rRNA genes were predicted by using MITOS tools (Bernt et al. 2013), and tRNAs were done through tRNAscan-SE webserver (Lowe and Chan 2016).

The complete mitogenome of this fly is circular and 15,384 bp in length (GenBank MH900079) and with weakly positive AT-skew (0.0180) and negative GC-skew (-0.1695). The base-pair composition is 39.48% for A, 13.12% for C, 9.31% for G, 38.09% for T, separately and A + T for 77.57%, obviously more than G + C for 22.43%. The whole mitogenome consists of 13 PCGs, 22 transfer RNA genes, 2 ribosomal RNA genes, and a non-coding region known as the CR (control region). J-strand codes 23 genes (*NAD2-3, NAD6, COX1-3, CYTB, ATP6, ATP8*, and 14 tRNAs), while N-strand codes 14 genes (*NAD1, NAD4, NAD4l, NAD5*, 8 tRNAs, and 2 rRNAs). The 37 genes arrangement is similar to the most common type of the putative ancestor of insects (Boore 1999; Cameron 2014).

To validate the position of this fly in the phylogenetic tree, the mitogenomes of 23 species in the family Tephritidae and the two outgroups, *Drosophila suzukii* and *D. melanogaster* (Diptera: Drosophilidae) were put together to construct maximum likelihood tree (ML) based on the General Time Reversible model in MEGA X software (Kumar et al. 2018). The tree inferred from 500 replicates was taken to represent the phylogeny of the species analyzed in this study. The result showed that *Acrotaeniostola dissimilis* of tribe Gastrozonini was a monophyletic branch and clearly separated from tribe Dacini and tribe Ceratitidini with significant bootstrap value supported (Figure 1). Furthermore, two subfamilies, Dacinae and Trypetinae, were correctly identified as assigned and monophyletic with high bootstrap confidence (Figure 1). In conclusion, the mitochondrial genome of *A. dissimilis* reduced in the present study can provide essential DNA molecular data for further phylogenetic and evolutionary analysis in family Tephritidae.

**Figure 1.**
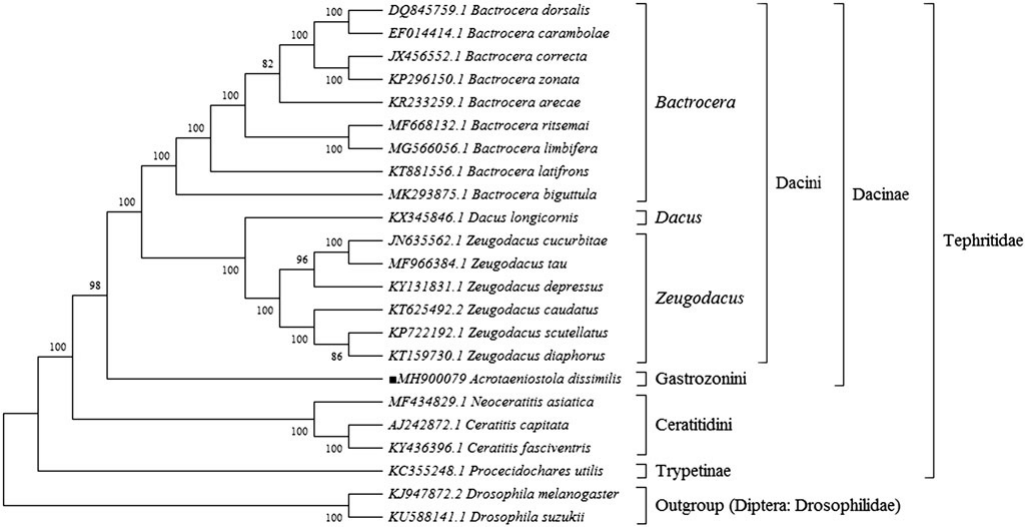
Molecular phylogeny based on complete mitogenome of the related 20 species in family Tephritidae and two outgroups. The position of *Acrotaeniostola dissimilis* is marked with solid square shape. Tree was constructed by maximum likelihood method with 500 bootstrap replicates. Genbank accession numbers lie before the scientific name of species.
